# Critical Enzymatic Functions of FTO in Obesity and Cancer

**DOI:** 10.3389/fendo.2018.00396

**Published:** 2018-07-30

**Authors:** Xiaolan Deng, Rui Su, Savanna Stanford, Jianjun Chen

**Affiliations:** ^1^Department of Systems Biology and The Gehr Family Center for Leukemia Research, Beckman Research Institute of City of Hope Monrovia, CA, United States; ^2^School of Pharmacy China Medical University, Shenyang, China

**Keywords:** fat mass and obesity-associated protein (FTO), obesity, cancer, mRNA *N*^6^-methyladenosine (m^6^A), m^6^A demethylase, AML, GBM, FTO inhibitors

## Abstract

Fat mass and obesity-associated protein (FTO) single-nucleotide polymorphisms (SNPs) have been linked to increased body mass and obesity in humans by genome-wide association studies (GWAS) since 2007. Although some recent studies suggest that the obesity-related SNPs in *FTO* influence obesity susceptibility likely through altering the expression of the adjacent genes such as *IRX3* and *RPGRIP1L*, rather than *FTO* itself, a solid link between the SNP risk genotype and the increased FTO expression in both human blood cells and fibroblasts has been reported. Moreover, multiple lines of evidence have demonstrated that FTO does play a critical role in the regulation of fat mass, adipogenesis, and body weight. Epidemiology studies also showed a strong association of FTO SNPs and overweight/obesity with increased risk of various types of cancers. As the first identified messenger RNA *N*^6^-methyladenosine (m^6^A) demethylase, FTO has been shown recently to play m^6^A-dependent roles in adipogenesis and tumorigenesis (especially in the development of leukemia and glioblastoma). Given the critical roles of FTO in cancers, the development of selective and effective inhibitors targeting FTO holds potential to treat cancers. This mini review discusses the roles and underlying molecular mechanisms of FTO in both obesity and cancers, and also summarizes recent advances in the development of FTO inhibitors.

## Introduction

As the first genome-wide association studies (GWAS)-identified obesity susceptibility gene, the fat mass and obesity-associated gene (FTO) has been well known for the strong association of the multiple single-nucleotide polymorphisms (SNPs) located in its intron 1 with risk of obesity (1–10). Although there are some controversial reports regarding the association between *FTO* SNPs and *FTO* expression (11–13), mouse model studies have shown the pivotal role of FTO in the regulation of fat mass, adipogenesis, and body weight (14–20). The link between the SNP risk genotype and increased *FTO* expression in human fibroblasts and blood cells has also been demonstrated (21–23). Studies have demonstrated that a strong association exists between *FTO* SNPs and/or overweight/obesity with the increased risk of various types of cancers (24–29), implying a role of FTO in the pathogenesis of cancers. Indeed, the oncogenic role of FTO has been reported in leukemia and glioblastoma (GBM), where FTO is highly expressed (30–32). More importantly, FTO was reported as the first *N*^6^-methyladenosine (m^6^A) demethylase of eukaryotic messenger RNA (mRNA) (33), and the functions of FTO in adipogenesis and tumorigenesis have been linked to its m^6^A demethylase activity (30–32, 34). As the most abundant internal modification in eukaryotic mRNAs, m^6^A usually occurs at the consensus motif of RRm^6^ACH ([G/A/U][G>A]m^6^AC[U>A>C]); enriched in 3′ untranslated region (UTR), gene coding regions, and especially near stop codons (35, 36). The m^6^A modification is deposited by the METTL3-METTL14-WTAP methyltransferase complex (i.e., writer) (37–39) and can be removed by m^6^A demethylases (i.e., erasers) such as FTO and ALKBH5 (33, 40). The m^6^A modification functions as a post-transcriptional modulator of gene expression by decreasing or increasing mRNA stability, or promoting mRNA translation efficiency through its recognition of different m^6^A reader proteins (41–48). The roles of m^6^A modification and the associated machinery in the pathogenesis of various types of cancers have been reported recently (30–32, 48–59). This review focuses on the functions of FTO in both adipogenesis and tumorigenesis and on the underlying m^6^A-dependent mechanisms, along with a brief discussion of recent advance in the development of FTO inhibitors and their therapeutic potential to treat cancers.

## Association of FTO with overweight/obesity and its role in adipogenesis

**Figure 1:**
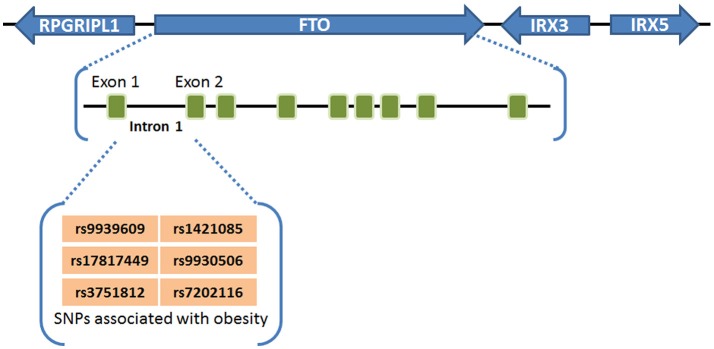
FTO SNPs associated with obesity. *FTO* SNPs in intron 1 (rs9939609, rs17817449, rs3751812, rs1421085, rs9930506, and rs7202116) have a strong association with overweight or obesity (61).

Obesity and overweight populations have become a global crisis, with the numbers increasing every year in adults and children. In 2015, there were 603 million adults and 108 million children who were diagnosed obese in 195 countries, and the population suffering with obesity has increased two-fold in over 70 countries during 25 years (60). Obesity is commonly caused by inherited or behavioral factors (food intake, physical activities, etc.), and it may induce other chronic diseases: diabetes, heart disease, chronic kidney disease, bone disorders, and many types of cancer (10, 26, 60). SNPs of *FTO* in intron 1 was first found to be associated with human obesity in European populations in 2007 (1–3), and subsequently validated by different groups in other populations including Asians (4–6), Africans (7), Hispanics (8), and Native Americans (9, 10), demonstrating a strong association between *FTO* SNPs in intron 1 (rs9939609, rs17817449, rs3751812, rs1421085, rs9930506, and rs7202116) and overweight or obesity (61) (see Figure 1). People carrying FTO risk alleles typically have a high body mass index (BMI), which may be due to a higher food intake (62, 63) and diminished food satiety (64), but not related to energy expenditure (62). Meta-analysis studies (65–67) have validated and confirmed that the influence of FTO variants on obesity risk is attenuated through physical activities as well as dietary and drug-based interventions (68, 69), although the underlying mechanism remains elusive. Some recent studies have suggested that the association between FTO SNPs in intron 1 and obesity might be owing to their potential influence on expression of *IRX3, IRX5*, and *RPGRIP1L*, rather than on their expression of *FTO* (11–13). However, there is also compelling evidence showing that such *FTO* SNPs are associated with increased expression of FTO (21–23, 70, 71). Moreover, animal model studies have shown that FTO plays a critical role in regulating fat mass, adipogenesis, and total body weight (14–20). For instance, FTO-deficient mice develop postnatal growth retardation and show a reduction in both adipose tissue and lean body mass (14). Conversely, overexpression of FTO in mice develops obesity by increased food intake (15), demonstrating the pivotal role of FTO expression itself in obesity (58). Therefore, there is no doubt that there is still a robust association of the FTO expression level/function with obesity and increased body mass, though the underlying mechanism has yet to be fully elucidated.

The recent discovery of FTO acting as an m^6^A eraser paved a novel way to reveal the molecular mechanism that links FTO with the increased susceptibility to overweight and obesity. A study in 2013 showed that the *FTO* obesity-risk allele (rs9939609 T/A) is associated with increased FTO expression, reduced m^6^A ghrelin mRNA methylation, and increased ghrelin expression (22). Ghrelin, the “hunger hormone,” is a key mediator of ingestive behavior, and its increased expression results in increased food intake and a preference for energy-dense foods, tending to lead to overweight and obesity (22, 72). A later study also reported that the *FTO* genotype (the AA (risk) genotype at the rs9939609 locus of *FTO*) impacts food intake and corticolimbic activation (73).

Excessive accumulation of adipose tissue under obese condition is a main mechanism for storage of excess energy (61). It has been reported that a positive correlation exists between the FTO level in subcutaneous adipose tissue and BMI, with a higher *FTO* mRNA level in adipose tissue from obese individuals than that in control populations (61, 74, 75). Zhao et al. demonstrated that FTO-mediated m^6^A demethylation regulates mRNA splicing and plays a critical role in the regulation of adipogenesis (34). They showed that *FTO* expression is inversely correlated with the m^6^A level during adipogenesis, and *FTO* depletion blocks differentiation and wild-type FTO (but not FTO mutant) restores adipogenesis; mechanistically, FTO mediates differentiation through the regulation of m^6^A levels around splice sites, thereby controlling the exonic splicing of the adipogenic regulator factor RUNX1T1 (34, 76). Similarly, another study also revealed that the demethylase activity of FTO is functionally required for pre-adipocyte (3T3-L1) differentiation (77). Furthermore, Merkestein et al. showed FTO regulates adipocyte differentiation *in vivo*, and further revealed that FTO enhances adipocyte numbers during mitotic clonal expansion at an early stage of adipogenesis (19). The compelling evidence of these studies supports FTO-mediated m^6^A demethylation playing a pivotal role on adipogenesis regulatory.

## Association of FTO with cancers and its oncogenic role in both tumorigenesis and drug response

Epidemiology studies show that FTO SNPs (including rs9939609, rs17817449, rs8050136, rs1477196, rs6499640, rs16953002, rs11075995, and rs1121980) and overweight/obesity are strongly associated with an increased risk of various types of cancers, including breast cancer, prostate cancer, kidney cancer, endometrial cancer, pancreatic cancers, lymphoma, and leukemia (24–29). For instance, several SNPs of intron 1 of FTO (including rs7206790, rs8047395, rs9939609, and rs1477196) are all significantly associated with breast cancer risk, and rs1477196 shows the strongest association (29). Notably, SNPs outside of intron 1 of FTO could also be associated with cancer risk. For example, rs16953002 of intron 8 of FTO has been identified to be significantly associated with melanoma risk (28). It is possible that the obesity-associated SNPs lead to increased expression of FTO, which in turn contributes (at least to some extent) to an increased susceptibility to overweight and obese, as well as an increased risk of cancer development (30). Indeed, several recent studies have suggested that FTO plays an oncogenic role in various types of cancers such as leukemia, brain tumor, breast cancer, gastric cancer, endometrial carcinoma, and cervical squamous cell carcinoma (CSCC) where it is overexpressed (30–32, 78–82). Li et al. provided the first *in vivo* animal model study demonstrating a critical oncogenic role of FTO in cancer (30). They reported that FTO is highly expressed in certain subtypes of acute myeloid leukemias (AMLs) such as those carrying t(11q23)/*MLL*-rearrangements, t(15;17)/*PML-RARA, FLT3*-ITD, and/or *NPM1* mutation (30). They further showed that forced expression of FTO significantly promoted human AML cell survival and proliferation and inhibited human AML cell differentiation and apoptosis, and forced expression of FTO significantly promoted leukemogenesis in mice (30). The opposite was true when endogenous expression of FTO was depleted (30). Subsequently, Su et al. reported that by the inhibition of FTO's oncogenic role, R-2-hydroxyglutarate (R-2HG), a previously well-recognized oncometabolite (83–90), actually exhibits a broad and intrinsic antitumor activity in AML and GBM (31). Cui et al. reported that targeting glioblastoma stem(-like) cells (GSCs) with a FTO inhibitor in mice could significantly inhibit the development of GSC-initiated tumor *in vivo* (32). It was also reported that the depletion of *FTO* expression significantly inhibited cell proliferation, migration, and invasion of human gastric cancer cell lines, and the opposite phenomenon was observed when FTO was forced expressed (80).

FTO has also been reported to affect the response of cancer cells to drug treatment. Li et al. showed that a knockdown of FTO could significantly enhance the response of human AML cells to all-trans retinoic acid (ATRA) treatment and promote ATRA-induced AML cell differentiation (30). Su et al. reported that analogous to FTO depletion, R-2HG treatment also sensitized human AML cells to standard chemotherapeutic agents such as ATRA, azacitidine (AZA), Decitabine, and Daunorubicin *in vitro* (31). They further showed that R-2HG treatment also sensitized human AML cells to Decitabine and Daunorubicin *in vivo* in immunodeficient xenotransplantation recipient mice (31). Similarly, Zhou et al. reported that FTO enhanced the resistance of CSCC cells to chemo-radiotherapy (82). Consistent with the function of FTO in drug resistance, it was reported that overexpression of FTO is a marker for poor prognosis in cancers such as gastric cancer and endometrial carcinoma (80, 81).

Mechanistically, the roles of FTO in tumorigenesis and drug response have been linked to its m^6^A demethylase activity. Li et al. reported that FTO negatively regulates expression of a set of tumor suppressor target genes, such as *ASB2* and *RARA* [two genes implicated in leukemia cell proliferation and drug response (91–93)], through post-transcriptionally modulating m^6^A abundance of the target mRNA transcripts and thereby affecting their stability (30). Su et al. further reported that FTO also positively regulates expression of a set of oncogenic targets such as *MYC* and *CEBPA* through an m^6^A-dependent mechanism (31). The suppression effect of the FTO inhibitor on GSC growth/proliferation and survival is also believed to be owing to the inhibition of the m^6^A demethylase activity of FTO (32). In CSCC, FTO has been reported to enhance chemo-radiotherapy both *in vitro* and *in vivo* through positively regulating expression of β-catenin (CTNNB1) via an m^6^A-dependent mechanism (82). Collectively, evidence is emerging that FTO plays critical oncogenic roles in various types of cancers as an m^6^A demethylase, and post-transcriptionally regulates expression of a number of functionally important target genes through m^6^A-dependent mechanisms.

## Identification of small molecule inhibitors targeting FTO

Since the discovery of FTO as an m^6^A demethylase in 2011 (33), efforts have been made to identify selective small-molecule inhibitors targeting FTO's m^6^A demethylase activity (94–98). FTO belongs to the AlkB family, and the crystal structure of FTO resolved in 2010 (99) shows a strong Fe (II) and α-ketoglutarate (αKG) dependent activity as a dioxygenase, at N-terminals. Chen et al. reported in 2012 that rhein, a natural product, competitively binds to an FTO active site, and exerts an inhibitory activity on FTO-dependent m^6^A demethylation in cells, through directly disrupting the bindings between FTO and the m^6^A substrate (94). In 2014, Zheng et al. developed a selective FTO inhibitor that also selectively inhibits the m^6^A demethylase activity of FTO and increases the m^6^A levels in cells (95); a later study showed that this FTO inhibitor (i.e., MO-I-500) could significantly inhibit the survival and/or colony formation of human SUM149 cells, a triple-negative inflammatory breast cancer cell line (97). Meclofenamic acid (MA), a nonsteroidal anti-inflammatory drug, was discovered to specifically inhibit FTO's m^6^A demethylase activity, while paring ALKBH5 (96). MA has been further proved to effectively inhibit the survival and growth of GBM cells through suppression of the m^6^A demethylase activity of FTO (32). In addition, Compound 12 has been developed based on a α-KG tethering strategy, which could selectively inhibit FTO over other AlkB subfamilies (including ALKBH5) and α-KG oxygenases (98). Su et al. showed that R-2HG is also an inhibitor of FTO that binds direct to FTO protein and significantly inhibits the m^6^A demethylase activity of FTO in a dose-dependent manner, leading to a significant increase of global m^6^A abundance in R-2HG-treated sensitive leukemia cells (31).

## Discussion and conclusions

**Figure 2:**
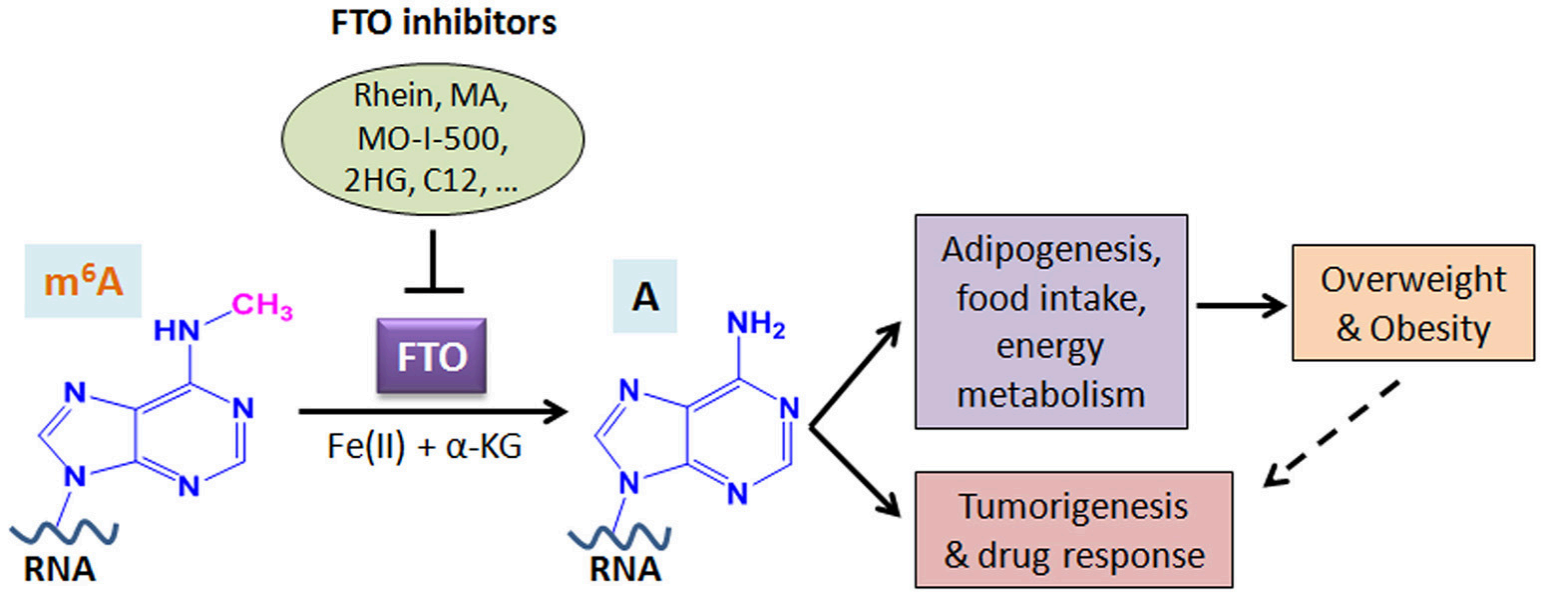
Schematic illustration of the roles of FTO in RNA m^6^A modification, overweight/obesity, and tumorigenesis/drug response. As an m^6^A demethylase, FTO post-transcriptionally regulates expression of its critical target genes and thereby contributes to overweight/obesity (likely through affecting adipogenesis, food intake, and energy metabolism) and cancers (including tumorigenesis and drug response). The obesity-associated function of FTO in metabolism may also contribute to cancers. Inhibition of FTO-mediated m^6^A demethylation by various inhibitors holds therapeutic potential to treat FTO-overexpressing cancers. MA, meclofenamic acid; 2HG, 2-hydroxyglutarate; C12, Compound 12 (98).

A growing body of evidence suggests that FTO plays critical roles in both overweight/obesity and cancers. As the first m^6^A demethylase identified, FTO has been shown to regulate expression of a number of important target genes through post-transcriptionally reducing their m^6^A levels and thereby affecting the stability and/or splicing of target mRNAs, in turn leading to promoting adipogenesis, tumorigenesis, and drug resistance of cancer cells. Therefore, although FTO may regulate expression of distinct sets of target mRNAs in different cell types, it affects overweight/obesity and cancers likely through similar, m^6^A demethylase activity-dependent mechanisms (see Figure 2). The strong association between FTO SNPs or overweight/obesity with an increased risk of cancers suggests that the obesity-associated function of FTO in metabolism may also contribute to its effects in cancers (Figure 2). Indeed, the FTO gene variant related to cancer risk is unlikely independent of adiposity (100). In addition, it was reported that by targeting the PI3K/AKT signaling, FTO influences breast cancer cell energy metabolism including lactic acid, ATP, pyruvate kinase activity, and hexokinase activity (79).

Given the essential role of FTO in cancer development and drug resistance, targeting FTO holds therapeutic potential in treating cancers in which FTO is overexpressed. Thus far, FTO inhibitors have been tested *in vitro* and *in vivo*, and show potent antitumor effects in treating both GBM and breast cancer (32, 97). Similarly, Su et al. showed that by targeting FTO directly, R-2HG exhibits a strong antitumor effect in both leukemia and GBM, especially when in combination with standard chemotherapeutic agents (31). These studies provide proof-of-concept evidence demonstrating that FTO is a realistic druggable target in treating cancers. In the near future, when more effective and selective inhibitors of FTO are developed, they could be applied, especially in combination with other therapeutic agents, into the clinic to treat various types of cancers. On the other hand, although FTO also plays a role in obesity, it was argued that FTO might not be a good pharmaceutical target to treat obesity, because the factors leading to obesity might be more complex (101, 102). Thus, a deeper understanding of the factors contributing to obesity could lead to the development of therapeutics targeting obesity.

## Author contributions

XD and JC drafted and revised the manuscript, while RS and SS contributed to the revision of the manuscript.

### Conflict of interest statement

A patent has been filed by JC and RS based on their work on R-2HG/FTO. The remaining authors declare that the research was conducted in the absence of any commercial or financial relationships that could be construed as a potential conflict of interest.

## Abbreviations

FTO: the fat mass and obesity-associated protein
SNP: single-nucleotide polymorphism
GWAS: genome-wide association study
mRNA: messenger RNA
m^6^A: *N*^6^-methyladenosine
GBM: glioblastoma
UTR: untranslated region
BMI: body mass index
CSCC: cervical squamous cell carcinoma
AML: acute myeloid leukemia
R-2HG: R-2-hydroxyglutarate
GSCs: glioblastoma stem(-like) cells
ATRA: all-trans-retinoic acid
AZA: azacitidine
αKG: α-ketoglutarate
MA: meclofenamic acid.
